# Distinct Chemical Cues Reprogram Cellular and Multicellular Phenotypes in Ovarian Cancer Spheroids

**DOI:** 10.1002/smll.202506120

**Published:** 2025-09-16

**Authors:** M Sreepadmanabh, Meenakshi Ganesh, Jimpi Langthasa, Ramray Bhat, Tapomoy Bhattacharjee

**Affiliations:** ^1^ National Centre for Biological Sciences Tata Institute of Fundamental Research Bangalore 560065 India; ^2^ Radiation Therapy Stanford University School of Medicine Stanford CA 94305 USA; ^3^ Department of Developmental Biology and Genetics Indian Institute of Science Bengaluru Karnataka 560012 India; ^4^ Centre for Bioengineering Indian Institute of Science Bengaluru Karnataka 560012 India

**Keywords:** biochemical regulation, cancer spheroids, chemical reprogramming, multicellular collectives, ovarian cancer

## Abstract

The self‐organization of cellular collectives is crucial in development and cancer. Multicellular aggregation in cancer is associated with a higher efficiency of metastasis. However, it is not fully understood how mechanochemical microenvironmental cues affect the organization and stability of such ensembles. Here, using a model system of ovarian cancer spheroids, which temporally transit from solid, dysmorphic moruloids to structurally plastic, lumen‐containing blastuloids, it is shown that the periodic volume fluctuations observed in blastuloids are driven by lumenal fluid influx and cell‐cell junctional states. Furthermore, blastuloid cell states are reprogrammed, which enables them to rapidly recover from even complete structural disintegration and self‐organize into fully lumenized ensembles. Using targeted chemical perturbations, two distinct cues are identified that regulate separate transition traits: calcium levels establish cell states cognate to, and pH regulates the fluctuation dynamics of blastuloid phenotypes. The work holds significant implications toward understanding mechanisms governing structural resilience and plasticity in complex cellular assemblies.

## Graphical abstract

Calcium and pH directly control both the physical state‐dependent cellular reprogramming and conformational plasticity of multicellular organization in ovarian cancer spheroids.

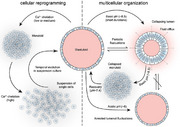

## 1 Introduction

Collective multicellular phenotypes exhibit a rich class of biological behavior distinct from their single‐celled constituents, such as self‐driven morphogenesis, structured spatial organization, and diverse functional roles.^[^ ^1^, ^2^, ^3^ ^]^ Investigating the determinants of these processes have important implications toward understanding multicellular evolution, pattern formation, and pathogenesis of cancer.^[^ ^4^, ^5^, ^6^, ^7^ ^]^ A classic example of cellular aggregates is spheroids—such as those formed by stem cells and cancer cells—which are commonly used as in vitro models for investigating development, stem cell biology, and cancer.^[^ ^5^, ^8^, ^9^ ^]^ Despite being a widely adopted experimental system, fundamental questions on their emergent biophysical properties remain elusive. For example, several types of spheroids exhibit progressive morphogenesis—what begins as a mass of aggregated cells eventually manifests structurally distinct physical states.^[^ ^10^, ^11^ ^]^ This is commonly observed in ovarian cancer, wherein, spheroids found in the ascitic fluid, despite their common epithelial origin, exhibit distinct morphologies—either solid morula‐like (“moruloid”) or hollow blastula‐like (“blastuloid”) masses.^[^ ^12^ ^]^ Whereas moruloids exhibit uneven surfaces and dysmorphic morphologies, blastuloids possess smooth surfaces, spheroidal shapes, and well‐defined lumens. These forms are more than mere aggregates of cells—rather, they achieve complex tissue‐like organization, with the blastuloid exhibiting a centrally positioned lumen surrounded by epitheloid cells and an enveloping extracellular matrix (ECM) sheath.^[^ ^13^ ^]^ Recent work on these systems has provided insights on how cell‐matrix interactions drive transitions of moruloids into blastuloids,^[^ ^12^ ^]^ as well as impart mechanical resilience to the blastuloid structures.^[^ ^14^ ^]^ While it is reasonable to expect that spheroids and other disseminated multicellular aggregates—which are suspended within circulatory fluid spaces and compartments—would be exposed to the chemical cues within them, the role of such cues toward the maintenance and stability of spheroidogenesis remains incompletely understood.

Here, we employ self‐aggregating epithelial‐origin ovarian cancer cells (OVCAR‐3), which are exemplars of a unicellular‐moruloid‐blastuloid transition. Interestingly, blastuloids demonstrate a time‐variant structure—we observe fluid flux‐driven alterations to the lumenal dimensions on an hours‐long time scale. Direct visualization of such lumenal fluctuations reveals a partial‐to‐complete collapse of the central cavity, followed by a progressive recovery. Biochemical assays implicate calcium‐dependent intercellular junctions as regulators of blastuloid stability, with the extent of structural recovery being strongly dependent on the dosage and duration of calcium chelation‐induced stress. We also observe that cells in the blastuloid undergo a conformational‐dependent state‐reprogramming, which imparts structural resilience as well as enables rapid recovery of fully‐lumenized blastuloids even after a complete disintegration of the original ensemble into single cells. However, we also show that extreme degrees of calcium stress completely reset this reprogramming. Hence, our work identifies calcium as a direct regulator of the individual cell state. We further go on to show that environmental pH separately exerts a strong influence on the multicellular states: while acidic pH stabilizes the blastuloids but abrogates lumenal fluctuations, basic pH forces a reversible blastuloid‐to‐moruloid transition. Cumulatively, our work identifies distinct chemical cues modulating separate conformation‐specific phenotypic traits, which together explain both the structural plasticity and the mechanical stability of cancer cell collectives.

## 2 Results

### 2.1 Multicellular OVCAR‐3 Aggregates undergo Temporal Evolution of Form, Resulting in Structurally Plastic Lumenized Ensembles

**Figure 1:**
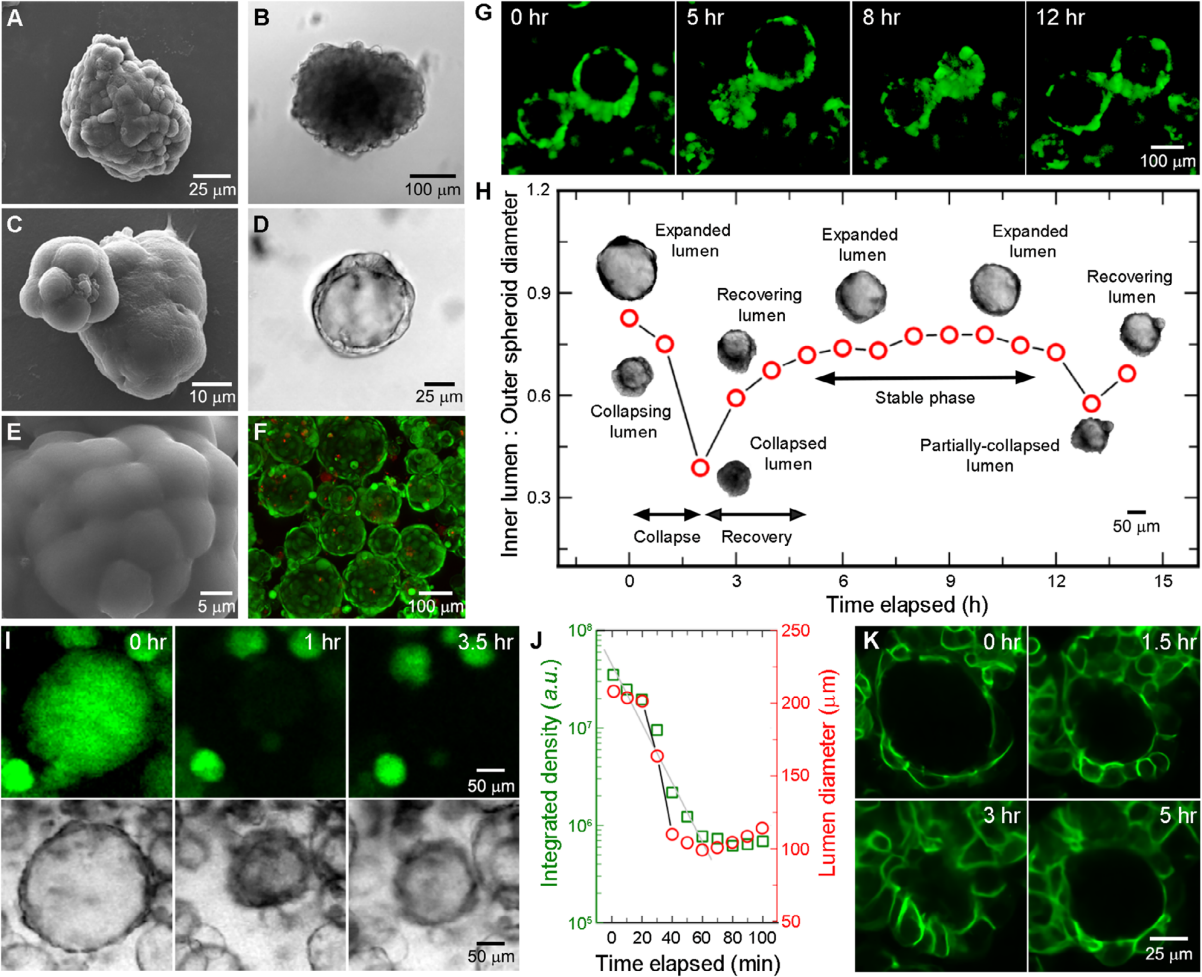
Scanning electron and brightfield micrographs showing A,B) a moruloid (solid cell mass) and C,D) a blastuloid (lumenized structure), formed by OVCAR‐3 cells grown in suspension. E) The smooth ECM coating formed around a mature blastuloid. F) Cell viability as assessed by staining with Calcein‐AM (green, marking viable cells) and propidium iodide (red, marking membrane‐compromised cells), indicating that lumenization is not driven by necrosis. G,H) Time‐lapse imaging and time trace quantification of the ratio between inner lumenal diameter and outer spheroidal diameter for blastuloidal structural transitions. Blastuloids composed of GFP‐expressing cells exhibit a periodic oscillation between distinct conformational states, characterized by a collapse of the fully lumenized structure leading to lumen compaction, which is followed by progressive expansion and recovery of the fully lumenized conformation. I,J) Tracking and quantifying fluid flow using fluorescein‐laden blastuloids implicates fluid flux as the mechanism driving periodic conformational transitions. K) Blastuloids composed of OVCAR‐3 cells engineered to constitutively express E‐cadherin tagged to GFP show a distinct change in cell shape and cell‐cell junctions during lumen collapse and recovery phases, suggesting that junctional stability is critical for such conformational transitions.

To enable self‐assembly of OVCAR‐3 cells into multicellular collectives, we employ non‐adherent suspension systems. While the cells are initially free‐floating and homogeneously dispersed, we observe a progressive aggregation that occurs over several days, resulting in well‐defined cellular masses, akin to solid morula‐like spheroids. These clusters begin to form within 24–48 h of seeding, followed by a progressive increase in size up until approximately 4–5 days, which we call a moruloid (**Figure** **1**A,B). Subsequently, moruloids develop a cavitation, whereby the formerly solid masses (i.e., having a cellular core) develop well‐defined hollow inner lumens resembling blastula‐like structures (Figure 1C,D). Whereas moruloids exhibit an uneven surface and dysmorphic structure, the lumenized spheroids—which we term blastuloids—develop a smooth ECM coating^[^ ^12^ ^]^ and appear almost perfectly spherical by day 9–10 (Figure 1E). Importantly, lumenization is accomplished not by any necrosis but by intraspheroidal intercellular movement,^[^ ^12^ ^]^ suggesting that these distinct conformational phenotypes are programmed evolutions of form (Figure 1F). Hence, under non‐adherent culture conditions, we observe a distinct temporal evolution of collective form—marked by single cells organizing into moruloid aggregates, which subsequently transition into lumenized blastuloids.

Surprisingly, while the basic blastuloidal form remains stable over time, using time‐lapse imaging, we find that they exhibit dramatic size oscillations between different conformational states (Figure 1G,H; Figure S1 and Video S1, Supporting Information). Remarkably, these transitions are highly reversible—best characterized by a clear decrease in the lumenal size through a sharp collapse, followed by a complete recovery of the original form. Even though similar structural oscillations have been previously reported,^[^ ^12^, ^15^, ^16^, ^17^, ^18^ ^]^ the underlying mechanism remains unclear. Here, we hypothesize that the lumenal space acts as a fluid filled‐sac and the transitions are enabled by fluid exchange with the external continuum. Collapsed lumenal states could be attributed to large contractile stresses exerted by the peripheral layer of cells,^[^ ^19^ ^]^ the imbalance between internal and external hydrodynamic pressure,^[^ ^20^, ^21^ ^]^ or a destabilization of the cell‐cell junctions holding the blastuloid together. Similarly, the recovery and stabilization are likely driven by fluid influx to the lumenal space, along with reinforcement of the cell‐cell junctions.

To test whether fluid exchange occurs between the extra‐lumenal and intra‐lumenal space during periodic conformational changes, we first incubate pre‐formed blastuloids in liquid media with added fluorescein. Following an overnight incubation—given the hours‐long time scale of structural oscillations—we collect the blastuloids by gentle centrifugation, wash off the excess fluorescein‐containing media, and resuspend these in fluorescein‐free media. Indeed, we observe that lumenal spaces appear fluorescent, indicating that fluid enters from the continuum into the lumens (Figure 1I). However, this uptake could also have been facilitated by the passive diffusion of fluorescein molecules through the intercellular spaces. Hence, to test whether lumenal fluid gets pumped back to the external environment during collapse, we perform time‐lapse imaging of such fluorescein‐laden blastuloids. Here, by quantifying the intra‐lumenal fluorescein intensity, we show that the lumenal collapse results in a specific loss of fluorescence, and subsequent transition to an expanded state does not recover the signal intensity—indicating a loss of intra‐lumenal fluorescein (Figure 1I,J). Together, these visual patterns support the idea that periodic conformational transitions in blastuloids are driven by a lumenal fluid influx from the extra‐spheroidal environment.

Despite this insight, the underlying physical mechanism enabling structural transitions in blastuloids remains elusive. To dissect this, we begin by considering what broad mechanical changes are required for a blastuloid to participate in ensemble conformational transformations—with the most likely contributor being cell‐cell adhesions mediated via epithelial cadherin (E‐cadherin)‐based junctions. Hence, we generate an OVCAR‐3 reporter cell line carrying a constitutively fluorescent fusion construct of green fluorescent protein (GFP) tagged to E‐Cadherin to visualize the junctional dynamics in blastuloids during conformational changes. Using this, we capture a striking pattern of progressive alterations in cell shape and junctional positions during specific states of the blastuloidal oscillations (Figure 1K; Video S2, Supporting Information). Specifically, we find that during the lumenal collapse, cells achieve columnar‐like morphology along the periphery; whereas during the lumenal expansion, cells assume a squamous morphology, compressed between the lumen and the outer ECM coat. Such altered organizations also impact the cell‐cell junctional arrangements, particularly emphasized by the difference between collapsed and the expanded lumens—while the former shows prominent interfaces between two adjacent cells, the latter feature a significantly reduced contact area between neighboring cells. Interestingly, we also find that the 3D nuclear morphology remains largely conserved during lumenal collapse (Figure S2, Supporting Information). Together, these observations strongly suggest that altered junctional arrangements underlie the conformational shifts exhibited by blastuloids. Motivated by this, we now direct our focus toward dissecting how junctional stability regulates the different structural states of such multicellular collectives.

### 2.2 Ca^2+^‐Dependent Junctional Stability Regulates Structural State Transitions and Conformational Stability of Blastuloids

**Figure 2:**
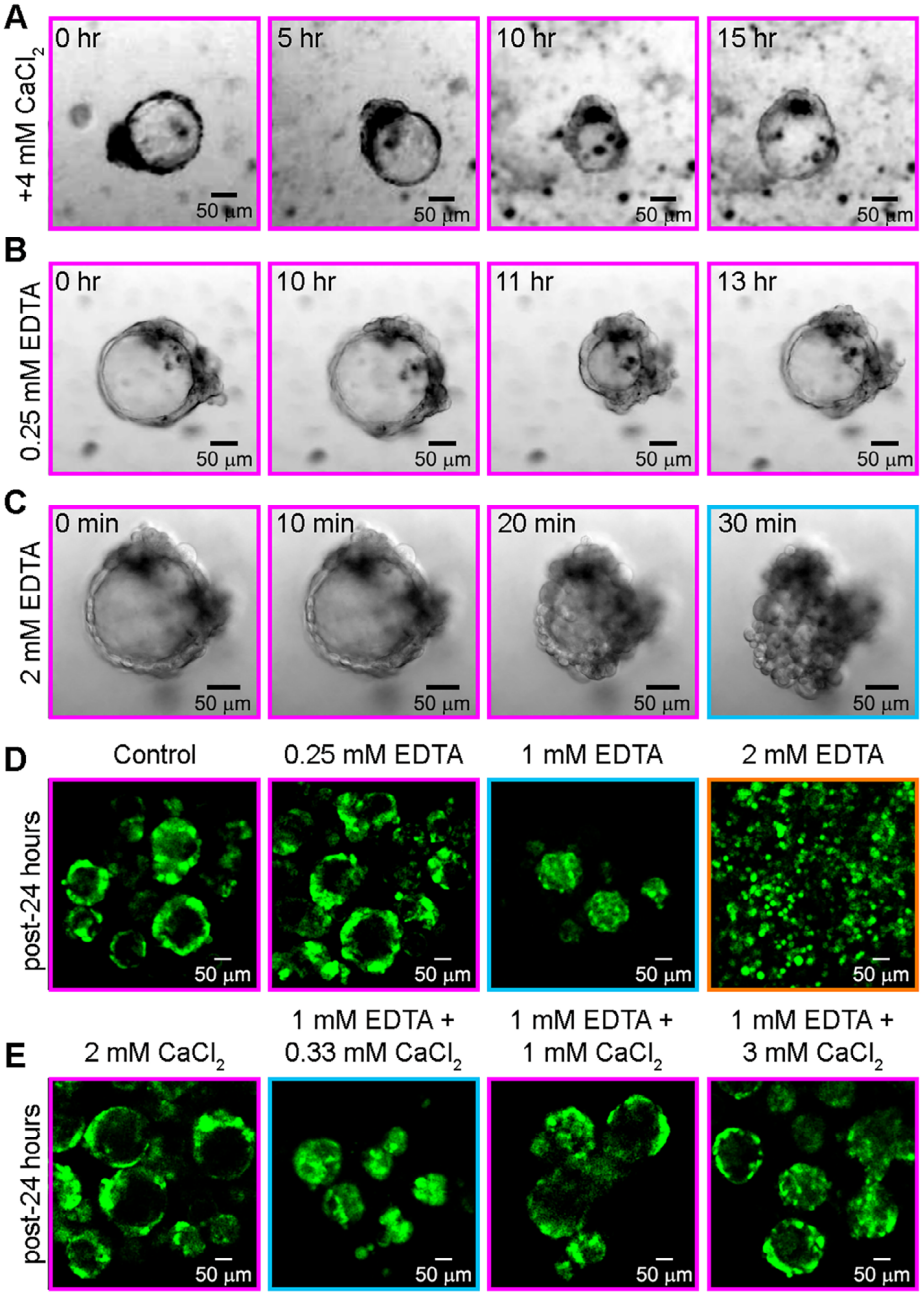
Neither (A) reinforcing the cell‐cell contacts via Ca^2+^ supplementation, nor (B) weakening the cell‐cell contacts using sub‐critical amounts of EDTA (Ca^2+^ chelation) significantly affects transitions between conformational states. C) High (2 mm) EDTA treatment heavily compromises cell‐cell contacts to completely destabilize blastuloids, causing a rapid collapse into moruloid‐like masses. D) Ca^2+^ chelation via EDTA treatment shows a stoichiometric effect on the blastuloid conformation, initially transitioning from fully lumenized to lumen‐compacted states, culminating in a completely disaggregated state—reminiscent of the single cell state prior to aggregation. E) Ca^2+^ supplementation directly counters the effects of EDTA treatment, showing that Ca^2+^‐dependent adhesion is specifically targeted by EDTA to engineer the observed C,D) conformational transitions. Color scale: orange indicates single cells, cyan indicates moruloids, and magenta indicates blastuloids.

E‐cadherin‐based cell‐cell junctions—such as those formed between OVCAR‐3 cells^[^ ^22^ ^]^ in a blastuloid—exhibit a well‐studied dependency on Ca^2+^, which enables inter‐domain binding.^[^ ^23^ ^]^ Calcium signaling has also been extensively implicated in cancer biology, especially as a determinant of metastatic potential.^[^ ^24^ ^]^ With specific regard to ovarian cancer, peritoneal calcium levels typically vary between <1 mm under physiologically healthy conditions to hypercalcemic levels (≈2.5 mm) in malignant ascites.^[^ ^25^, ^26^, ^27^ ^]^ Parallelly, past work has shown how Ca^2+^ chelation structurally destabilizes multicellular ovarian cancer spheroids as well as impairs their metastatic potential.^[^ ^28^, ^29^ ^]^ Hence, it is pertinent to ask whether and how variations in Ca^2+^ levels influence the structural state transitions and conformational stability of OVCAR‐3 ensembles. Stoichiometric analyses have previously established well‐defined concentration ranges across which Ca^2+^ can effectively mediate the stabilization of cadherin‐based cell junctions. The range of Ca^2+^ for such functions is 1–2 mm Ca^2+^; below 0.5 mm, a destabilizing effect is expected due to insufficient Ca^2+^ ion availability.^[^ ^30^, ^31^ ^]^ Thus, we first hypothesize that modulating the levels of Ca^2+^ in the system should directly (de)stabilize cell‐cell junctions, in turn affecting the blastuloid's conformational transitions. First, we subject the blastuloids to an elevated level of Ca^2+^ availability (+4 mm) by external CaCl_2_ supplementation. However, blastuloids continue to exhibit lumenal collapse and recovery, indicating that elevated Ca^2+^ levels do not affect conformational fluctuations in blastuloids (**Figure** **2**A). Next, we test whether lowering the Ca^2+^ availability may alter these conformational fluctuations. We achieve this using Ethylenediaminetetraacetic Acid (EDTA)‐based chelation (0.25 mm EDTA), which binds and sequesters Ca^2+^ ions from the media. Interestingly, we find no discernible effects on the structural transitions, which continue to cycle through the typical lumenal collapse and recovery pattern (Figure 2B; Figure S3, Supporting Information).

While these observations might appear contrary to our hypothesis that cell‐cell adhesions modulate conformational plasticity, it may be speculated that destabilizing the complex multicellular blastuloid organization requires a higher degree of perturbation, given that components such as the ECM coat could bring in additional mechanical reinforcement via cell‐matrix interactions^[^ ^32^, ^33^, ^34^ ^]^ (as is indeed the case, which we demonstrate in Figure S4, Supporting Information). Hence, we exacerbate the degree of Ca^2+^ deprivation by chelation using 2 mm EDTA. This immediately manifests a catastrophic structural failure, resulting in a complete breakdown of the blastuloid form (Figure 2C; Video S3, Supporting Information). This collapse not only obliterates the lumen, but also effects a blastuloid‐to‐moruloid‐like structural transition. Intrigued by this, we immediately revisit and expand upon prior experiments by applying different degrees of EDTA‐based Ca^2+^ chelation to blastuloids, and indeed find a stoichiometry‐dependent structural response (Figure 2D). Specifically, we report that following 24 h of treatment, sub‐critical levels (≤0.33 mm EDTA) of Ca^2+^ chelation do not effect a significant alteration to the blastuloid conformation. However, elevating this to 1 mm EDTA engineers a pronounced lumenal collapse, resulting in moruloid‐like ensembles—similar to the blastuloid‐to‐moruloid transition observed over short treatment time scales (<30 min) using 2 mm EDTA. And finally, exposing blastuloids to 24 h of high dosage (2 mm) EDTA treatment triggers a complete disaggregation into single cells, reminiscent of the initial starting point for the entire cycle spanning temporal evolution of collective form. We show that these effects are not due to loss of cell viability as a result of elevated salt stress, but rather a loss of mechanical organization following the chemical perturbation (Figure S5, Supporting Information).

We also test for the specificity of these phenotypes to Ca^2+^ dysregulation, by directly countering the destabilizing effects of EDTA using a stabilizing supplementation of CaCl_2_ over 24 h of treatment. Indeed, we find that a balance between the antagonistic effects of these chemical cues combinatorially regulates different conformational states of the blastuloids. We observe that when the destabilizing agent (EDTA) is stoichiometrically higher than the stabilizing agent (CaCl_2_) the blastuloids exhibit a lumenal collapse, followed by a blastuloid‐to‐moruloid transition. Conversely, when the destabilizing agent is stoichiometrically lesser than, or equivalent to, the stabilizing agent, the blastuloid architecture remains conserved (Figure 2E). Together, these findings position Ca^2+^‐dependent junctional stability as a potent regulator of structural state transitions and conformational stability in organized multicellular collectives.

### 2.3 Ca^2+^ Controls Both the Blastuloidal State‐Induced Cellular Reprogramming and Conformational Plasticity of Multicellular Collectives

In addition to establishing Ca^2+^‐mediated cell junctional stability as a direct determinant of physical organization in OVCAR‐3 spheroids, the experiments applying Ca^2+^ chelation also raise an interesting question concerning the perturbed morphologies—how reversible are the conformational changes induced by EDTA treatments? This is particularly interesting in light of the temporal evolution of form exhibited by moruloids maturing into blastuloids. While moruloid formation from single cells takes 3–4 days, the subsequent cavitation process to form blastuloids takes an additional 4–5 days. This transition from single cells to moruloids to blastuloids proceeds through a reprogramming of cell state.^[^ ^12^ ^]^ However, as we observed using targeted chemical perturbations, EDTA treatment over a minutes‐long time scale proves sufficient to disrupt highly organized blastuloid conformations that evolve over days. This mismatch between the timescales of disruption versus formation leads us to pose three fundamental questions. Do cells retain a “memory” of their collective organizations? To what extent does such structural perturbation alter the cellular state? And, is the persistence of “memory” subject to the degree of perturbation?

**Figure 3:**
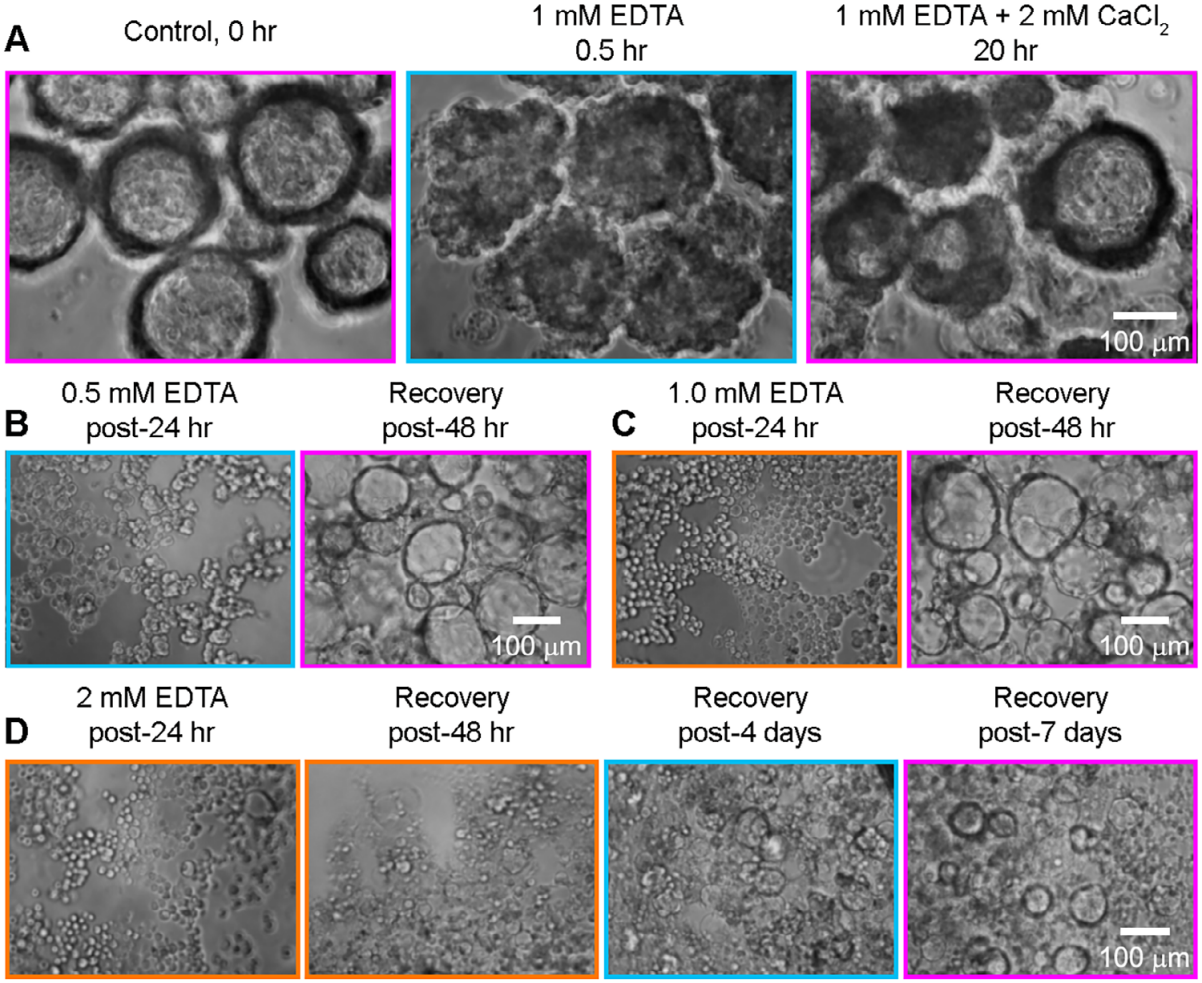
A) Reversing EDTA‐induced blastuloid‐to‐moruloid transition using Ca^2+^ supplementation, suggesting that conformational states are plastic and that effects from a destabilizing perturbation (EDTA) can be overcome by a stronger stabilizing agent (CaCl_2_). B,C) Low and medium dosage (0.5 and 1 mm) EDTA treatments over prolonged durations (24 h) induces a blastuloid‐to‐unicellular transition. However, upon removal of the destabilizing stress, these single cells revert and organize into fully lumenized blastuloid structures within 48 h. D) A heavy dosage (2 mm) EDTA treatment over a prolonged duration (24 h) also induces a blastuloid‐to‐unicellular transition. However, these cells now require 7 days to form the lumenized blastuloid structure. Hence, disrupting the Ca^2+^‐dependent cell‐cell adhesion in multicellular OVCAR‐3 collectives affects both conformational transitions and cellular reprogramming, depending on the magnitude and duration of the destabilizing perturbation. Color scale: orange indicates single cells, cyan indicates moruloids, and magenta indicates blastuloids.

To systematically interrogate these aspects, we begin by subjecting well‐lumenized 10‐day‐old blastuloids to an intermediate grade of EDTA treatment (1 mm) for a brief duration of 30 min. This proves sufficient to effect a blastuloid‐to‐moruloid transition – albeit, without complete disaggregation into single cells (**Figure** **3**A). Remarkably enough, rescuing this with the addition of 2 mm CaCl_2_ (stoichiometric excess over the EDTA levels) enables a significant recovery of lumenized blastuloid phenotype within <24 h (Figure 3A). Such accelerated recovery is particularly notable, given that the standard moruloid‐to‐blastuloid transition occurs over several days. These results strongly suggest that cells organized within specific collective forms retain a state‐specific “memory” (ostensibly via altered gene expression) which is retained or at the least allows them to quickly recover the state even upon dissociation.

Next, to explore the extent to which structural perturbations alter the cellular state, we expose blastuloids to progressively stronger EDTA treatments—low (0.5 mm), intermediate (1.0 mm), and high (2.0 mm)—over long durations (24 h). These combinations prove sufficient to ensure a complete disaggregation of fully‐lumenized blastuloidal structures into constituent single cells. All signs of lumenized conformations are lost, and effectively, all three combinations achieve a blastuloid‐to‐unicellular transition (Figure 3B–D). Surprisingly, removal of this Ca^2+^ chelation stress allows cells exposed to both low and intermediate‐degree EDTA treatments to revert to their original blastuloidal state, complete with well‐formed lumens, all within 48 h. In contrast, the entire unicellular‐to‐blastuloid transition takes around 7–10 days under the normal course of suspension culture. These rapid recovery dynamics imply that structured collective forms impart a resilient reprogramming to the constituent cells, which enables robust re‐organization into the original conformation following destabilizing stresses.

However, the high‐degree EDTA treatment provides an interesting exception: herein, the single cells disaggregated from the blastuloids after EDTA treatment get reset to a normal course of suspension culture—they accomplish moruloid‐like states within 4 days, while exhibiting lumenization by day 7. Therefore, in contrast to the prior cases of low and intermediate‐degree EDTA perturbations, there appears to be no acceleration of the blastuloid development timeline as a consequence of the cells’ past history. Rather, a high enough dosage and long enough duration of Ca^2+^ chelation effectively resets the cells to their native state, erasing all structural memory of the blastuloidal conformation. These observations together establish the boundaries of conformational resilience and plasticity of state transitions, both of which remain operational within quantifiable physical limits.

**Figure 4:**
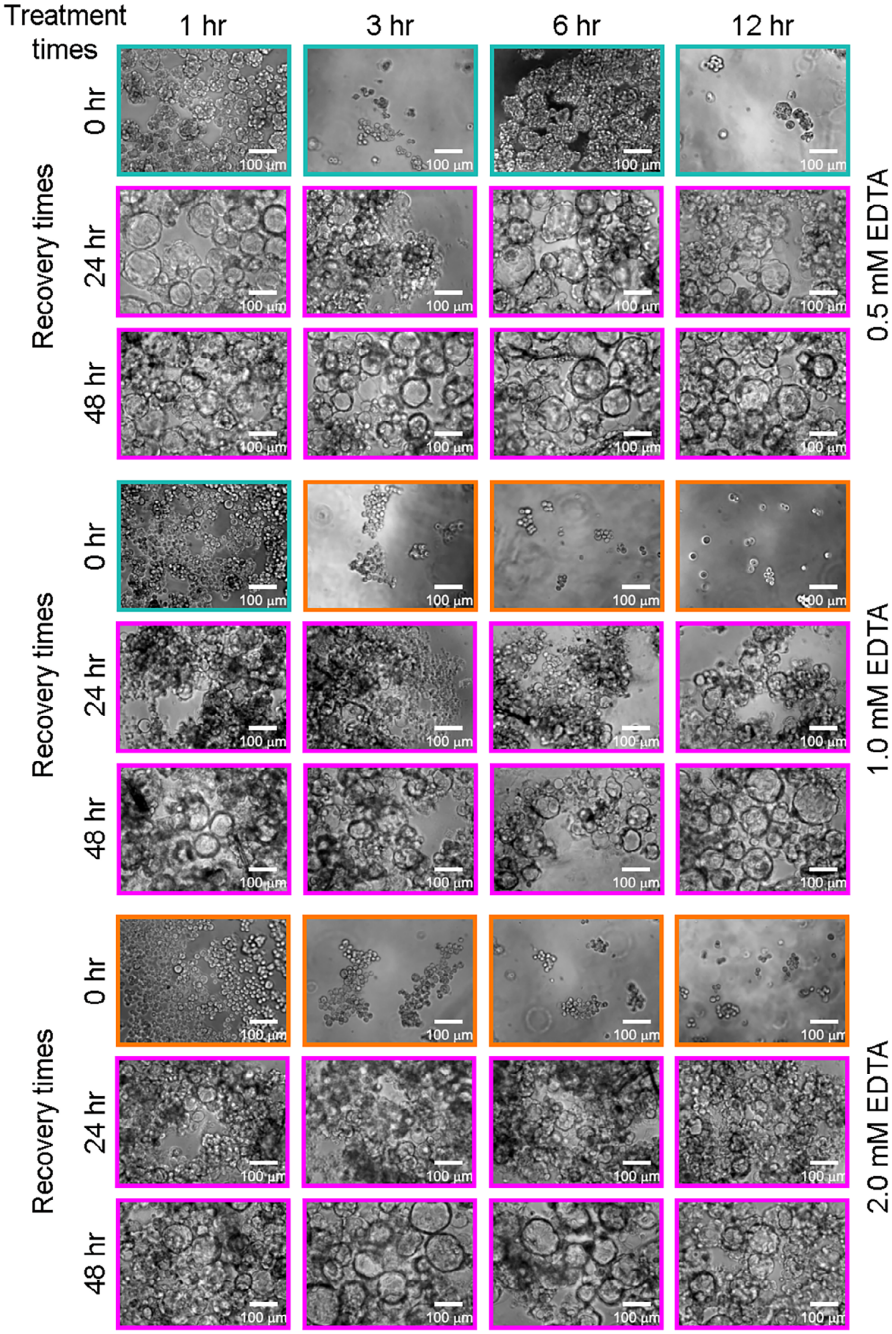
Destabilizing Ca^2+^ chelation stress—applied via EDTA treatments across different dosages and different treatment times—causes both blastuloid‐to‐moruloid and blastuloid‐to‐unicellular transitions. Further assessment at different recovery time points shows that reversion to stable lumenized states is possible from various (but not all) starting conformations. Hence, conformational plasticity and recovery potential is directly dependent on Ca^2+^‐based cell‐cell adhesion, which in turn regulates the individual cellular state. Color scale: orange indicates single cells, cyan indicates moruloids, and magenta indicates blastuloids.

Building on the limits established above, we now explore how conformational plasticity depends on the degree of perturbation—specifically with respect to both the dosage and duration of the Ca^2+^ chelation stress. In addition to subjecting well‐lumenized blastuloids to varying degrees of EDTA treatments—low (0.5 mm), intermediate (1.0 mm), and high (2.0 mm)—we simultaneously vary the duration of exposure, ranging from 1 to 12 h. Here, we profile the systems at two different recovery times of both 24 and 48 h post‐cessation of the respective EDTA treatment (**Figure** **4**). Briefly, we observe that across all tested treatment times, low‐degree EDTA stresses are most easily overcome, with a reversion to the blastuloidal state within as little as 24 h. However, both intermediate and high‐degree stresses require extended recovery periods up till 48 h to fully re‐form lumenized structures. It is worth noting that since the high‐degree treatment is only applied for 12 h as opposed to 24 h, here, the cellular state does not get fully reset, allowing disaggregated cells to recover the blastuloidal phenotype within 48 h. Together, these experiments generate a comprehensive map of structural transitions, which establishes that the conformational plasticity existing between single cells, moruloids, and blastuloids is indeed dependent on both the degree and duration of Ca^2+^ chelation. Importantly, from all these results, we infer that Ca^2+^‐driven regulation of junctional stability is a potent regulator of the individual cell state, driving the emergence of structurally‐distinct collective organizations.

### 2.4 pH Regulates the Multicellular State

Beyond Ca^2+^‐induced changes, it is interesting to consider yet another broadly varying and influential environmental variable – pH. Specifically, and with regard to ovarian cancer, the chemical properties of ascitic fluid exhibit pH‐dependent variations. Patient data suggests that the peritoneal microenvironment is largely basic in nature.^[^ ^26^, ^27^, ^35^ ^]^ However, this can shift toward a more acidic pH during ovarian cancer progression,^[^ ^36^ ^]^ spontaneous bacterial peritonitis, and cirrhosis^[^ ^37^, ^38^ ^]^—indeed, an acidic ascitic fluid pH shows strong correlation with mortality.^[^ ^38^ ^]^ Localized acidification is also commonly associated with tumor microenvironments as a consequence of metabolic reprogramming.^[^ ^39^ ^]^ In this regard, prior work using ovarian,^[^ ^40^ ^]^ lung,^[^ ^41^ ^]^ and breast^[^ ^42^ ^]^ cancer cell lines have also reported acidic pH‐induced upregulation of metastatic potential. In particular, acidic pH exerts an inhibitory effect on ovarian cancer cell proliferation, which mirrors the temporary dormancy achieved by suspension‐cultured spheroids via reversible c‐MYC expression.^[^ ^43^ ^]^ Hence, acidic pH potentially acts as a microenvironmental regulator aiding metastasis and chemotherapeutic evasion during circulation.

Interestingly, pH also regulates junctional integrity. Biochemical analysis of cadherin‐cadherin binding shows that low (acidic) pH promotes dimerization, leading to mechanically reinforced complexes that remain stable even in a Ca^2+^‐independent manner.^[^ ^23^, ^44^ ^]^ These reports, combined with our own findings, led us to speculate that environmental pH could potentially act as a direct regulator of the different structural transitions exhibited by OVCAR‐3 spheroids. Hence, we hypothesize that while acidic pH might be expected to stabilize the cell‐cell junctions and thereby the blastuloidal conformation, basic pH may possibly destabilize the ensemble structure. Here, we subject the blastuloids to acidic and basic fluid microenvironments by altering the medium pH between 6 and 8.5. Our cell viability measurements show that within this range cellular health is not compromised, ensuring any observed effects are due to loss of organizational structure and not cell death (Figure S6, Supporting Information).

**Figure 5:**
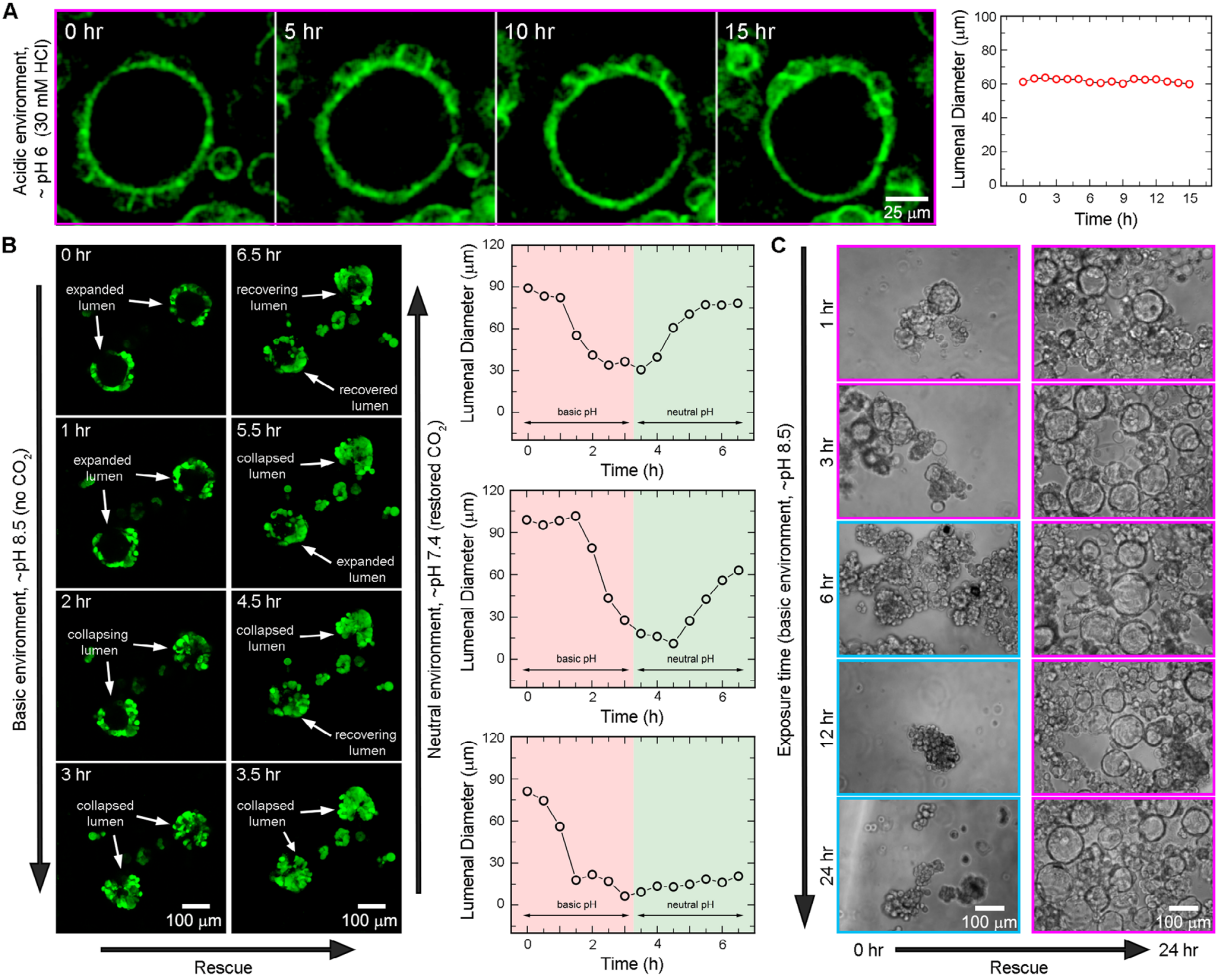
A) Exposure to acidic pH stabilizes the blastuloid conformation, arresting it in a fully lumenized state and abrogating the periodic transitions between different lumenal conformations. B) Exposure to basic pH destabilizes the fully lumenized blastuloid conformation, triggering a collapse that results in a moruloid‐like state. These effects are reversible, since a neutral pH environment enables recovery of the lumenized state. C) The blastuloid‐to‐moruloid transition effected by basic pH are plastic, and moruloids formed by different durations of basic pH treatment all accomplish a complete reversion to the fully lumenized blastuloidal state under neutral environmental pH. Color scale: cyan indicates moruloids and magenta indicates blastuloids.

We investigate the effects of acidic pH on blastuloidal structure by chemically adjusting the media pH to ≈6 by external addition of 30 mm HCl (**Figure** **5**A; Figure S7A and Video S4, Supporting Information). Here, we observe a remarkable stabilization of the blastuloidal conformation, with the lumenal dimensions remaining intact over at least 15 h. Indeed, the acidic pH seemingly freezes the blastuloid in a fully‐lumenized state, and completely abrogates the periodic conformational transitions. Given that acidic environments stabilize cadherin dimers, we conclude that lowered pH arrests the lumenal collapse process via mechanically reinforced cell–cell junctions.

**Figure 6:**
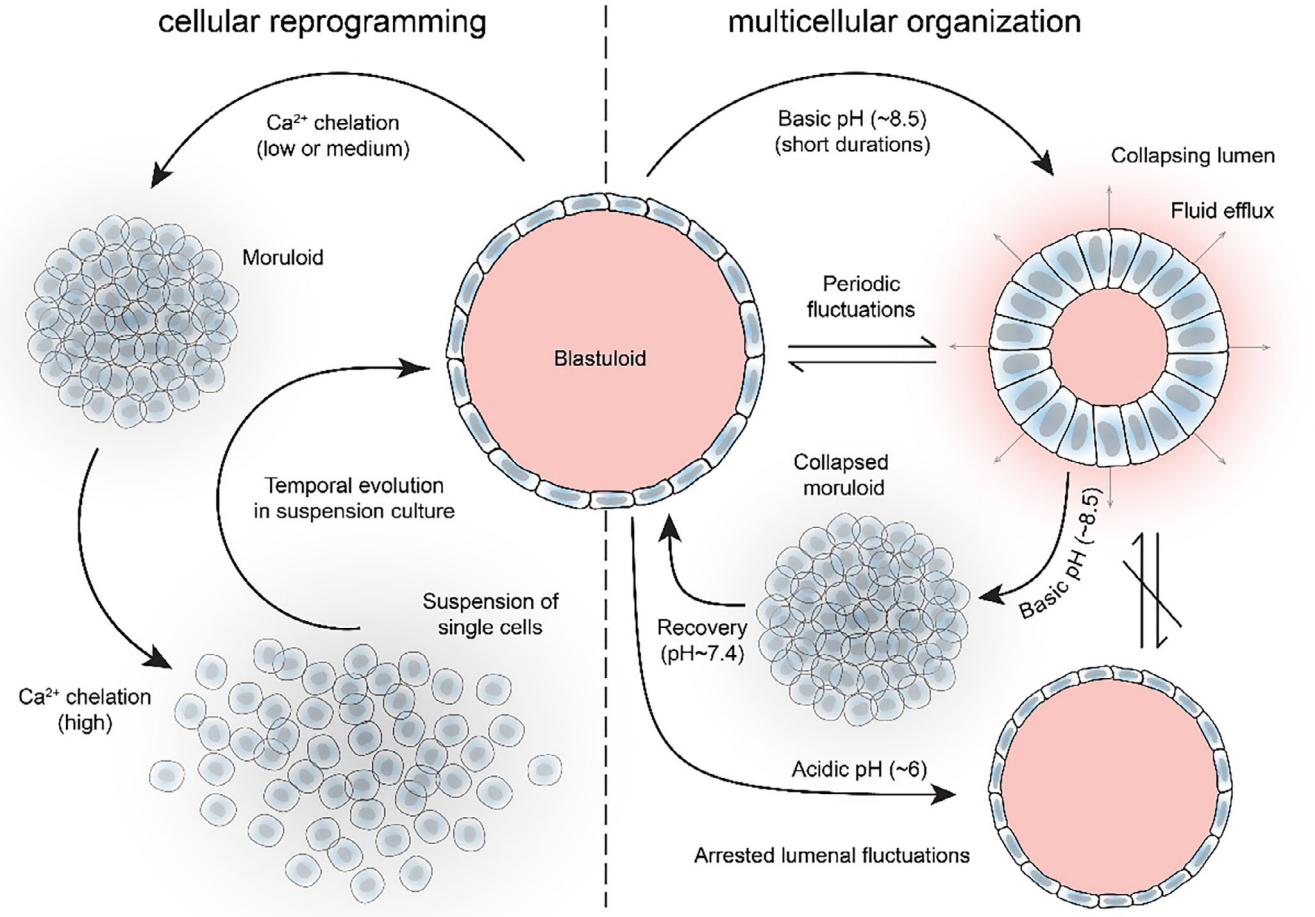
Calcium and pH control morphological state transitions in ovarian cancer spheroids, by regulating the individual and collective cell state, respectively.

Finally, by subjecting intact blastuloids to basic pH, we immediately find observations attesting to a destabilizing effect (Figure 5B; Figure S7B and Video S5, Supporting Information). Here, the degree of basic pH is retained at ∼ pH 8.5, which is achieved by cutting off the CO_2_ supply to the culturing environment—rapidly elevating the media pH. Basic pH effects a blastuloid‐to‐moruloid transition, without proceeding all the way to complete disaggregation of cells. These destabilizing effects are reversible, as resumption of the CO_2_ supply enables recovery of the blastuloidal structure at pH ∼ 7.4 (Figure 5B; Figure S7B and Video S5, Supporting Information). To test whether pH‐based regulation is indeed restricted to only the multicellular forms or extends all the way to the unicellular state, we next apply the basic pH stress over different durations up till a maximum of 24 h (Figure 5C). Interestingly, we do not observe any significant transition past the moruloid stage, which itself requires ≈3 h of exposure. Upon replenishing the CO_2_ supply after 6, 12, and 24 h of basic pH treatments, we find that lumenization is recovered, with moruloids reverting back to the blastuloidal state. Here, the recovery of blastuloidal state—unlike the Ca^2+^ chelation treatments—does not depend on the exposure time, indicating that pH only affects the collective cellular organization, without reprogramming the cell state. Together, our findings show that while basic pH destabilizes cell‐cell junctions and triggers a blastuloid‐to‐moruloid transition, acidic pH directly counters this by stabilizing the cell‐cell junctions and arresting lumenal fluctuations. Hence, while Ca^2+^ regulates the individual cell state underlying collective organizations and their structural transitions, pH gates the conformational switches between distinct multicellular states (**Figure** **6**).

## 3 Discussion

The observations from our study, when contextualized with respect to ovarian cancer metastasis, opens up several intriguing inquiries. A long‐standing question is the requirement for such phenotypically distinct forms of collective organization to coexist. Do these have functional consequences on the disease pathology? In addition, does the temporally evolved blastuloid form represent, in some sense, a more effective mediator for metastasis? Our observations of conformational plasticity, biochemical regulation of structural transitions, and spatial organization‐dependent cell state together allow framing of cogent hypotheses which will undoubtedly guide future investigations.

Recent reports on the rheological behavior of patient‐derived moruloids and blastuloids^[^ ^14^ ^]^ have documented that mechanical deformations—such as those expected during transit through the constraining and tortuous interiors of the peritoneum^[^ ^45^ ^]^—have markedly different effects on both conformations. Here, the structural resilience of blastuloids to mechanical shear appears significantly higher than that of moruloids—which has been attributed to the cell‐matrix interactions. In this regard, two separate observations from our current work stand out – one, that using chemical stressors (Ca^2+^ chelation), we arrive at similar conclusions of blastuloids being a more physically stable conformation, and one which can be recovered even after significant structural perturbations; and two, that a combined disruption of cell‐cell and cell‐matrix interactions also destabilizes the blastuloidal conformation in an additive manner. The latter observation is of direct pertinence and allows us to propose that the physical resilience exhibited by cancer cell collectives is a product of both the cell–cell and cell‐matrix interactions, and thus follows the morphogenetic logic employed in development. Furthermore, in the present work we have applied varying chemical perturbations while maintaining a constant physical milieu (free liquid). However, metastasizing cells typically traverse complex 3D niches with diverse material properties^[^ ^46^ ^]^—indeed, past work provides compelling evidence for a mechanical regulation of cancer progression.^[^ ^47^, ^48^ ^]^ We propose that future work may leverage 3D culture platforms that mimic the structural and viscoelastic properties of jammed and granular environs such as tissues.^[^ ^49^, ^50^ ^]^ Such constructs offer the capability to investigate how chemically uniform, yet mechanically heterogeneous 3D microenvironments^[^ ^51^ ^]^ influence living systems. Here, we demonstrate a proof‐of‐concept, illustrating how jammed packings of micron‐scale hydrogel particles can be employed to generate soft, granular, and self‐healing 3D matrices,^[^ ^52^ ^]^ suitable for studying dynamic ensemble‐scale phenomena such as the periodic conformational shifts in OVCAR‐3 blastuloids (Video S6, Supporting Information).

But what physical constraints force the emergence of such context‐dependent states? A possible explanation lies in observing single cell‐level rearrangements within moruloids and blastuloids, as reported previously.^[^ ^12^ ^]^ While cells in moruloid clusters undergo significant spatial rearrangements, blastuloidal cells appear largely locked in position. Structurally, this would seem apt – given that the blastuloid state forces cells into a jammed conformation surrounding a central lumen, affording little room for randomized movements that might otherwise jeopardize the ensemble structure. However, we hypothesize that such restrictions may also force cells to develop specialized temporally stable interfaces by triggering polarity‐inducing mechanisms. The stability of such interfaces and omic states would persist for limited periods even after disaggregation. This likely explains why recovery of lumenized structures proceeds at a remarkably rapid pace even after complete dispersion into single cells, provided the destabilizing perturbation is maintained within limits: as we have shown, while low degrees of Ca^2+^ chelation stress only structurally destabilize, high degrees of stress end up resetting the cell state, suggesting how even such a state persistence is contextual upon the chemical microenvironment. Importantly, this also reinforces the idea of a spatial position‐driven reprogramming which biases blastuloid‐derived cells toward recovering the lumenized conformation, rather than follow the standard unicellular‐moruloid‐blastuloid trajectory.

From both a fundamental and translational perspective, there is considerable value in determining the relative frequencies and rate constants associated with these state transitions. Such metrics would provide a quantitative basis toward modeling pathological progression and gauging the efficacy of therapeutic interventions. However, we recognize the technical challenges associated with such efforts, considering the complexity of the experimental system. First, accurate estimation of cell numbers is a considerable challenge, given both the altered (and presently unknown) proliferative dynamics of cells in the 3D spheroidal conformation as well as the days‐long time scales of these phenomena. This is further compounded by a small fraction of cells being likely shed off due to cell death, stochastic unbinding, or fluid shear. The dynamic nature of the fluidic milieus—both the in vitro suspension cultures and the in vivo ascites—introduces an additional complexity toward spatially‐resolved tracking and sampling of individual aggregates and single cells. As a first step, future work toward overcoming these limitations could make use of extensive and iterative sampling using a combination of proliferative markers, stable cell labeling, automated counting, and long‐term live imaging to generate statistically meaningful coarse‐grained estimates. Subsequent efforts could build on this by devising culture platforms across physiologically‐relevant volumetric scales to understand how density and system size alter these dynamics. This can be further improved by incorporating microfluidic setups with tunable flow rates that mimic the tortuous geometry of peritoneal cavities, in order to study how fluid shear influences the kinetics of structural transitions.

From a pathological perspective, it is important to consider the spectrum of phenotypic heterogeneities that typically manifest during ovarian cancer. Here, we have also investigated how cells corresponding to biologically‐distinct states respond to the chemical regulatory modules discussed in this work. For this, we employ two high‐grade serous carcinoma cell lines, which complement the OVCAR‐3: the COV362, a human ovarian epithelial‐endometroid carcinoma cell line derived from pleural fluid, and the OVCAR‐4, a multi‐drug‐resistant human ovarian epithelial carcinoma cell line derived from malignant ascites (similar to the OVCAR‐3′s origin).^[^ ^53^ ^]^ Biologically, the COV362 represents a much more mesenchymal phenotype as opposed to both OVCAR‐3 and OVCAR‐4 – likely a consequence of its BRCA1 mutation or MYC amplification, which facilitates epithelial‐to‐mesenchymal transition.^[^ ^53^, ^54^ ^]^ This has functional consequences for collective organization, since the mesenchymal nature of COV362 could impede the formation of ensemble structures requiring polarized cellular organisation (such as blastuloids). The OVCAR‐4, on the other hand, presents a somewhat intermediate stage between epithelial and mesenchymal modes;^[^ ^55^ ^]^ whereas, the OVCAR‐3 exhibits the most pronounced epithelial nature^[^ ^56^ ^]^—rendering it most likely to form lumenized ensembles. These differences are also reflected in prior evidence, wherein, both COV362 and OVCAR‐4 have been reported to form solid aggregates,^[^ ^12^, ^57^ ^]^ whereas, OVCAR‐3 aggregates readily mature into spatially‐organized blastuloids. Together, these three cell lines enable us to interrogate how chemical control over structural state and stability manifests across heterogeneous pathologically‐relevant morphologies.

Specifically, we first perform time‐lapse imaging of OVCAR‐4 collectives (Figure S8, Supporting Information), which shows that despite a lack of lumenization, the individual cells within these aggregates are highly dynamic, and continually undergo localized rearrangements (Video S7, Supporting Information). However, cells physically remain within the ensemble, as we do not observe any loss in the overall structural integrity, nor any significant expulsion of cells from the parent mass over hours‐long imaging timescales. These observations are also in agreement with prior reports^[^ ^12^ ^]^ that individual cells within moruloids exist in a more structurally dynamic and flexible regime, as opposed to blastuloid cells, which exist as “locked‐in” conformations. Next, by imposing calcium signaling stress on both COV362 and OVCAR4 aggregates (Figure S9, Supporting Information), we show direct parallels with the OVCAR3, wherein calcium deprivation triggers structural collapse. Indeed, whereas elevated calcium levels (via CaCl_2_ supplementation) do not elicit any discernible phenotypical differences, chelation of calcium (via EDTA treatment) exhibits a pronounced effect on the aggregates’ morphology, resulting in a complete collapse and dispersion of well‐defined 3D masses into dissociated single cell suspensions. Importantly, we also demonstrate that the effects of calcium stress are reversible (Figure S10, Supporting Information), and aggregate‐derived dissociated single cells go on to recover their original collective organisation once the perturbing chemical stress is removed. We also verify that such recovered structures retain excellent cell viability—implying that calcium stress‐induced structural collapse is not an experimental artefact arising due to cell death. Rather, perturbations to calcium signaling‐mediated cell‐cell adhesion underlie morphological transitions from self‐organized ensemble collectives to unicellular states—illustrating chemical control over structural organization.

Considering that complex cellular collectives such as blastuloids are largely observed in a metastatic scenario, it is also interesting to contextualize our findings toward explaining why the blastuloidal state is prevalent in the malignant ascitic environment. Undertaking large‐scale cellular rearrangements, forcing cells into jammed conformations, and maintaining fluid‐filled lumens requires heavy resource utilization—rendering the whole transformation from moruloid to blastuloid an energetically expensive process. Does the blastuloidal state confer benefits significant enough to waive off such considerations? An elementary argument could be that the periodic fluid flux helps avoid the classic nutrient‐depleted core that is an inevitable hallmark of most solid spheroidal aggregates. However, as has also been previously speculated, the spheroidal conformations^[^ ^58^ ^]^ and fluid flux might also provide potential resistance mechanisms against drug treatments by acting as efflux pumps. However, given both the hours‐long time scale of these conformational switches and that cells routinely cultured in undrugged culture media also exhibit the moruloid‐to‐blastuloid transition, it is difficult to consider this functionality as a strong enough pressure. Rather, we propose that the blastuloidal state renders the cellular ensemble physically robust enough to survive traversal through mechano‐chemically heterogeneous physiological niches. As shown here and from prior work,^[^ ^14^ ^]^ not only are blastuloids more resistant to mechanical deformations, they also exhibit a strong recoverability against destabilizing chemical cues. Consequently, these properties together confer an enhanced resilience against environmental perturbations, thereby improving the chances for successful metastatic dissemination.

In conjunction with prior work, our findings here advance the systematic characterization of a remarkably interlinked framework of mechano‐chemically tunable, interconvertible conformations, wherein both the individual and multicellular states are governed by distinct regulatory modules. Consequently, we envision that this structurally labile, temporally dynamic experimental system of OVCAR‐3 cellular collectives holds tremendous potential for exploring fundamental questions concerning morphogenesis, mechanobiology, and multicellularity.

## 4 Experimental Section

### Cell Culture and Spheroid Formation in Suspension Culture

Epithelial‐origin OVCAR‐3 cells are cultured in high‐glucose RPMI media supplemented with 20% fetal bovine serum and 1% penicillin‐streptomycin. Regular culturing for maintenance was performed on tissue culture‐grade plastic T‐25 flasks. The suspension cultures were set up by first generating a low‐adhesion surface, which involves using a 1% agarose solution to coat the base of a 35 mm plastic dish. Once the agarose polymerizes, the OVCAR‐3 cells are harvested from the 2D adherent culture by trypsinization and homogeneously disperse cells in the suspension culture dish. For seeding suspension cultures, between 150 000 and 200 000 cells were typically seeded per sample. Except where specifically mentioned, all cultures are performed using OVCAR‐3 cells constitutively expressing cytoplasmic GFP. The culture media was replenished at regular intervals (∼every 3 days) to get rid of cell‐generated debris. For this, the cellular clusters were gently spun down at low centrifugal speeds (500xg) for 5 min, which did not pellet down single or lysed cells. The resultant solid pellet was gently resuspended in nutrient media and reseeded into the agarose‐coated suspension culture dish. For experiments, the study iteratively retrieved small numbers (≈5–10) of blastuloids and transfer these to separate receptacles for the experimental assays. Here, it was ensured that mechanical shear did not deform the blastuloids by using pipette tips with the ends cut‐off to generate a wider nozzle. All blastuloids used in the assays exhibit well‐formed lumens prior to start of experiment. Both COV362 and OVCAR‐4 cells were cultured in a manner similar to OVCAR‐3 as described above. Solid spheroidal aggregates formed by both these cell lines were obtained following 3–4 days of suspension culture.

### CDH1‐GFP Tagged Overexpression in OVCAR3 Cell Line via Lentiviral Transduction

To generate CDH1‐overexpressing OVCAR3 cells, full‐length CDH1 cDNA was amplified by PCR from the untransformed MeT‐5A cell line using the following primers: E‐cadherin‐pCDH‐YFP forward primer, 5′‐actctagagctagcgaattcgccaccatgggcccttggagccgcagc‐3′; E‐cadherin‐pCDH‐YFP reverse primer, 5′‐ctttgctcaccattccactggatccgtcgtcctcgccgcctccg‐3′. The amplified product was cloned into a lentiviral expression vector (pCDH‐YFP backbone) using the Gibson Assembly method. Lentiviral particles harboring CDH1 cDNA, shRNA, or scrambled control constructs were generated by co‐transfecting the expression plasmid with packaging plasmids psPAX2 and pMD2.G (a kind gift from Dr. Deepak K. Saini) into 293FT cells (R70007; Thermo Fisher Scientific) using TurboFect transfection reagent (R0533; Thermo Fisher Scientific). Cells were cultured in DMEM supplemented with 10% fetal bovine serum (FBS). Conditioned medium containing lentiviral particles was collected at 48‐ and 72‐h post‐transfection, filtered through a 0.45‐µm syringe filter, and concentrated using the Lenti‐X Concentrator (631 232; TaKaRa) as per the manufacturer's instructions. Concentrated viral preparations were aliquoted and stored at −80 °C until further use. For transduction, OVCAR3 cells were seeded in 24‐well plates at 50–60% confluence and infected with lentiviral particles in the presence of 4 µg mL^−1^ polybrene for 24 h. After 72 h, transduced cells were selected with 5 µg mL^−1^ puromycin (CMS8861; HiMedia). Overexpression of CDH1 was confirmed by quantitative real‐time PCR (qRT‐PCR).

### Image Acquisition

Images were acquired using both a point‐scanning laser confocal microscope (Nikon A1R HD25) as well as a brightfield phase contrast ring‐fitted microscope (Nikon Eclipse). For confocal imaging, all samples were prepared in glass‐bottom dishes, whereas for the brightfield imaging, both glass and plastic‐bottom dishes were used. All live‐cell imaging was performed under controlled environmental conditions, with the temperature being maintained at 37 °C and the CO_2_ levels varied between either 5% (regular conditions) and 0% (to induce basic pH stress). All fixed timepoint imaging was performed with reference to either the experimental start time or the rescue start time, i.e., the treatment times indicate the time elapsed since addition of the chemical stress; the rescue/recovery times indicate the time elapsed since removal of the chemical stress and replenishment with fresh media.

### Fluid Flux Tracking with Fluorescein

To track the fluid flux when the blastuloidal lumen undergoes collapse and recovery, the water‐soluble dye fluorescein was employed. Briefly, blastuloids with well‐formed lumens are incubated overnight (≈12 h) in nutrient media supplemented with fluorescein. Following this, the blastuloids were collected as described above, pellet them down by centrifugation, and wash off the excess dye. These blastuloids were subsequently resuspended in unlabeled fresh nutrient media for time‐lapse imaging. To quantify the intra‐lumenal fluorescence intensity, the lumenal outlines were traced in ImageJ, and the in‐built functions were used to measure both the lumenal diameter as well as integrated density.

### Chemical Treatments—EDTA, CaCl_2_, HCl, and Collagenase

All chemical treatments were performed in complete nutrient media prepared as described above. For EDTA treatments, all dilutions were directly performed in nutrient media from a 0.5 m stock solution of EDTA prepared in double distilled water and sterile filtered via a 0.22‐micron syringe filter. All calcium chloride treatments were directly performed in nutrient media from a 1 m stock solution prepared in double‐distilled water and sterile filtered via a 0.22‐micron syringe filter. Both the 30 and 60 mm HCl treatments were applied by directly diluting a stock 12 m solution of concentrated HCl in nutrient media. Type I collagenase was reconstituted to a final concentration of 10 mg ml^−1^ and used at the specified concentration, directly in the nutrient media.

### Cell Viability Assays

To assess cell viability, calcein‐AM and propidium iodide were used to mark viable and dead cells, respectively. Calcein‐AM is a cell permeable small molecule that gets the fluorescence‐quenching AM moiety cleaved by esterase activity within live cells—thereby marking all live cells green. Propidium iodide fluoresces in red upon intercalation with DNA. However, it only enters the cells with compromised membranes—indicative of dead/dying cells.

### Quantification of Lumenal Dimensions

For quantifying the lumenal dimensions over time, brightfield imaging of blastuloids suspended in liquid media was used. Here, the lumenal outline was manually traced using the circular region select option in ImageJ, the dimensions of which were measured using built‐in particle analysis functions. For each time point and each blastuloid, both the inner lumenal diameter and the external spheroid diameter were measured. For the acidic and basic pH treatments, the lumenal diameter was measured directly. These values, as well as other measurements derived using these, were plotted for all such blastuloids across all times.

### Analysis of Nuclear Morphometrics Using 3D Segmentation and Tracking

Blastuloids in suspension cultures are nuclear‐stained with Hoechst (10 µm working concentration), followed by live cell confocal microscopy to obtain volumetric z‐stack images. Built‐in functionalities from the scikit‐image and simpleitk‐image‐processing toolboxes were employed using the open‐source Napari environment to accomplish post‐processing, segmentation, labeling, and morphometric quantification of 3D‐reconstructed nuclei for all time points. Following this, custom MATLAB codes were used to track the centroids of identified nuclei across all time points, and accordingly sort the overall morphometry data in the form of time traces corresponding to each individual nucleus.

### Scanning Electron Microscopy

To obtain SEM images of moruloids and blastuloids, an overnight fixation was performed using 2.5% glutaraldehyde, followed by three washes with PBS buffer for 5 min each to remove the unreacted fixative agent. Subsequently, the samples were washed five times with water for salt removal. Post‐washing, samples were subject to dehydration using varying concentrations of ethanol (30%, 50%, 70%, 90%, and 100%), and deposited on acid‐treated (1N HCl) coverslips, followed by air‐drying at room temperature.

## Conflict of Interest

The authors declare no conflict of interest.

## Author Contributions

T.B., R.B., and M.S. conceived the study. M.S. and T.B. developed the methodology with critical help from R.B. M.S. performed all experiments with help from M.G. J.L. performed the SEM experiments. M.S. analyzed all data with help from M.G. M.S. validated key findings and curated all data. M.S. with help from T.B. prepared the figures. M.S. wrote the manuscript with feedback from T.B. and R.B. R.B. contributed to the cell lines and culturing protocols. T.B. acquired primary funding support and supervised the overall project.

## Supporting information



Supporting Information

Supplemental Video 1

Supplemental Video 2

Supplemental Video 3

Supplemental Video 4

Supplemental Video 5

Supplemental Video 6

Supplemental Video 7

## Data Availability

The data that support the findings of this study are available from the corresponding author upon reasonable request.
