# Supplementary material for: Distinct Chemical Cues Reprogram Cellular and Multicellular Phenotypes in Ovarian Cancer Spheroids
Source: Small. 2025 Sep 16;21(44):e06120. doi: 10.1002/smll.202506120 (PMC12590526; doi:10.1002/smll.202506120)
Supplement: Supplementary file 1 — Supporting Information [file SMLL-21-e06120-s005.pdf]

NANO · MICRO
small

Supporting Information

for *Small*, DOI 10.1002/smll.202506120

Distinct Chemical Cues Reprogram Cellular and Multicellular Phenotypes in Ovarian Cancer Spheroids

*M Sreepadmanabh, Meenakshi Ganesh, Jimpi Langthasa, Ramray Bhat and Tapomoy Bhattacharjee**

Supplementary Information for

Distinct chemical cues reprogram cellular and multicellular phenotypes in ovarian cancer spheroids

M Sreepadmanabh[1], Meenakshi Ganesh[1], Jimpi Langthasa[2,3], Ramray Bhat[3,4], and Tapomoy Bhattacharjee[1,*]

[1] National Centre for Biological Sciences, Tata Institute of Fundamental Research, Bangalore, 560065, India

[2] Radiation Therapy, Stanford University School of Medicine, Stanford, California, 94305, USA

[3] Department of Developmental Biology and Genetics, Indian Institute of Science, Bengaluru, Karnataka, 560012, India

[4] Centre for Bioengineering, Indian Institute of Science, Bengaluru, Karnataka, 560012, India

[*] to whom correspondence should be addressed: tapa@ncbs.res.in

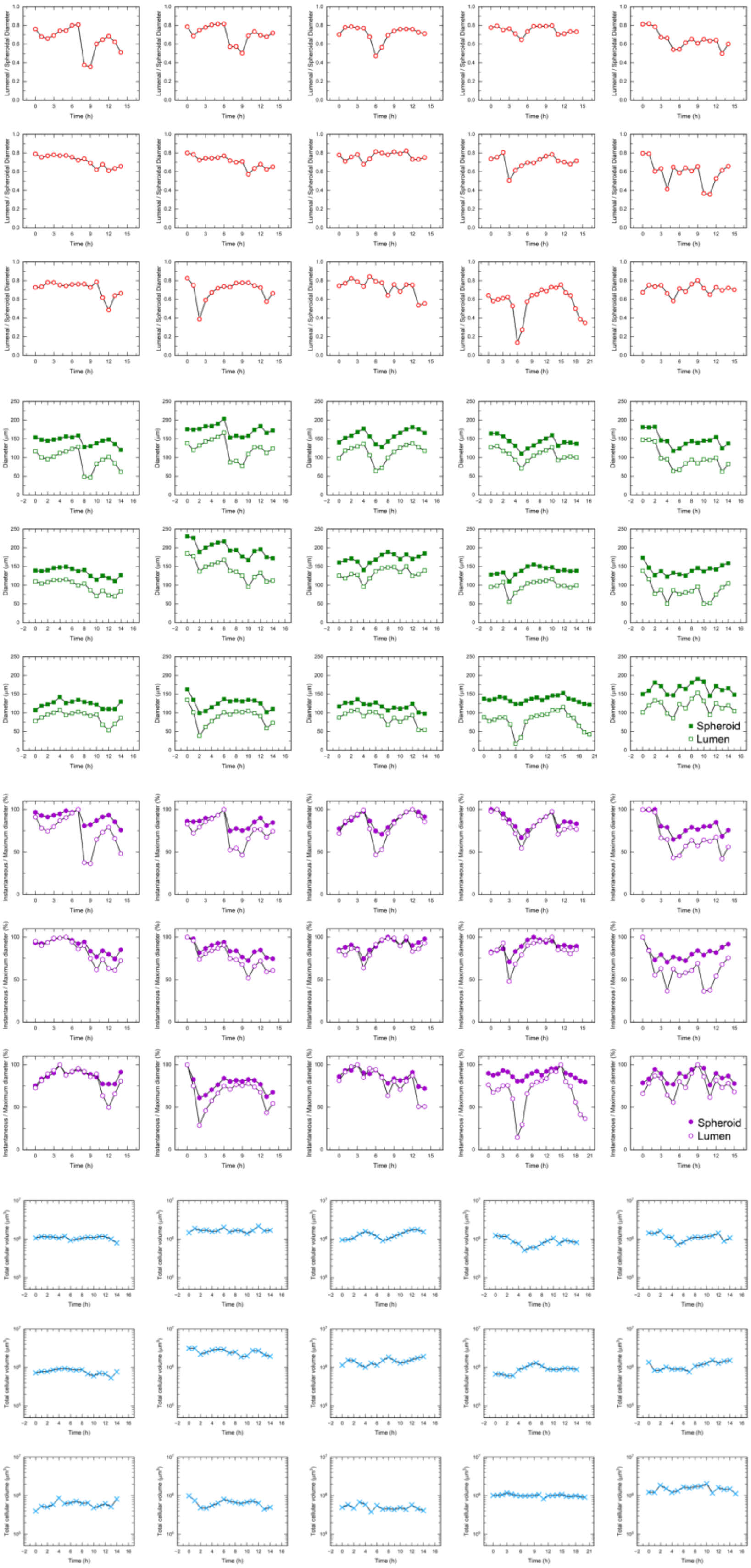

SI Fig. 1. Time trace analyses of the inner lumenal diameter and outer spheroidal diameter for blastuloids undergoing periodic transitions in conformational states while freely suspended in liquid media.

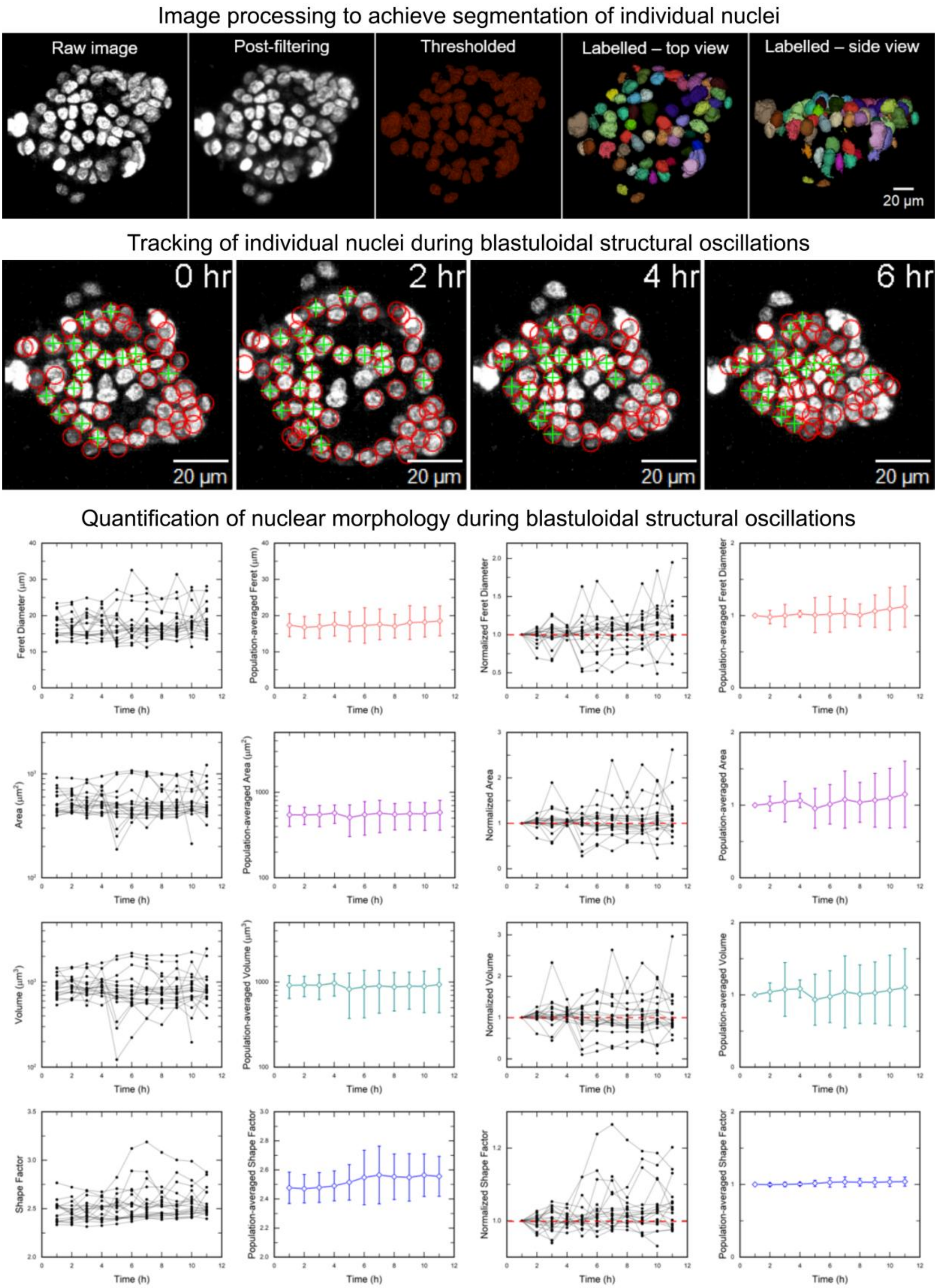

SI Fig. 2. 3D segmentation, tracking, and temporally-resolved morphometric dynamics of individual nuclei within a blastuloid undergoing one cycle of a periodic lumenal collapse.

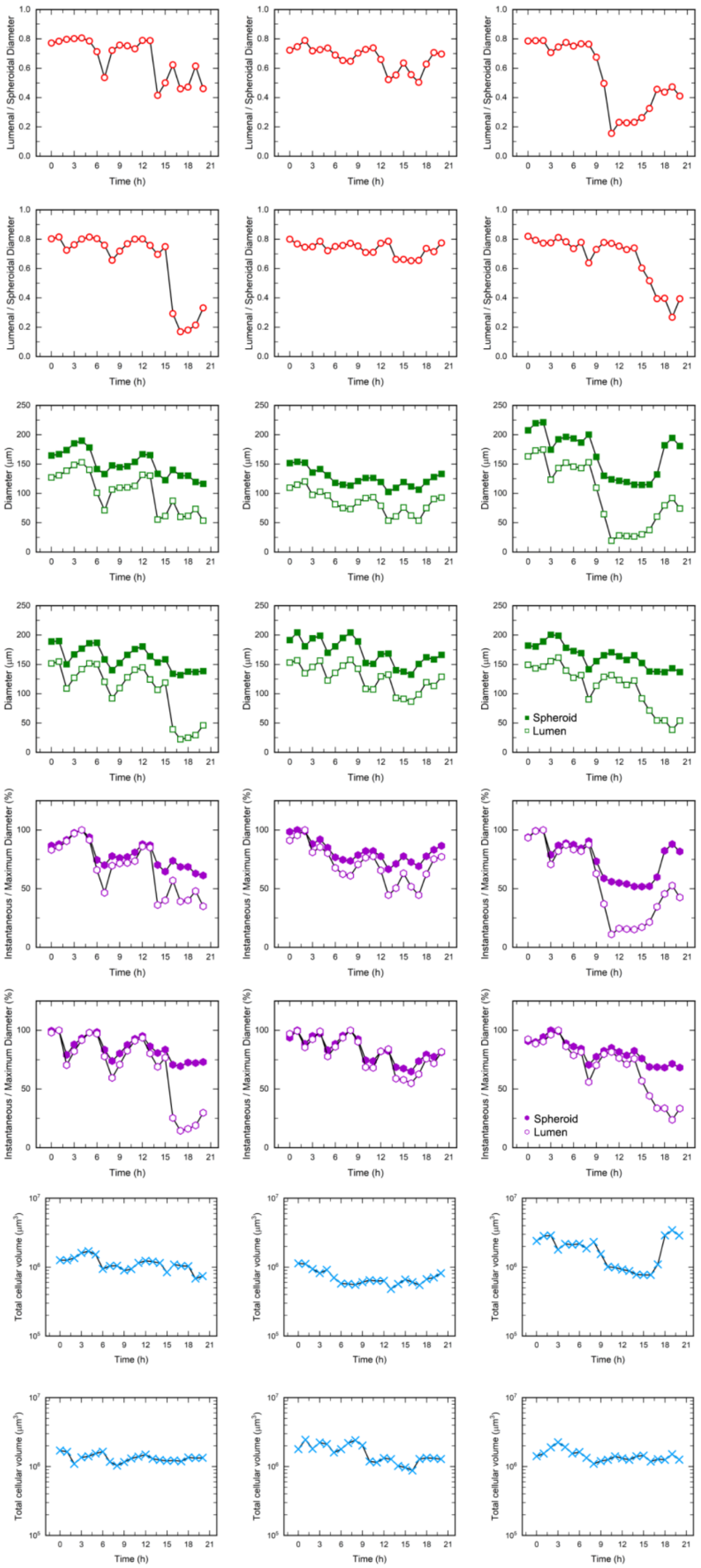

SI Fig. 3. Time trace analyses of the lumenal and outer spheroidal dimensions for blastuloids treated with 0.25 mM EDTA in liquid suspension.

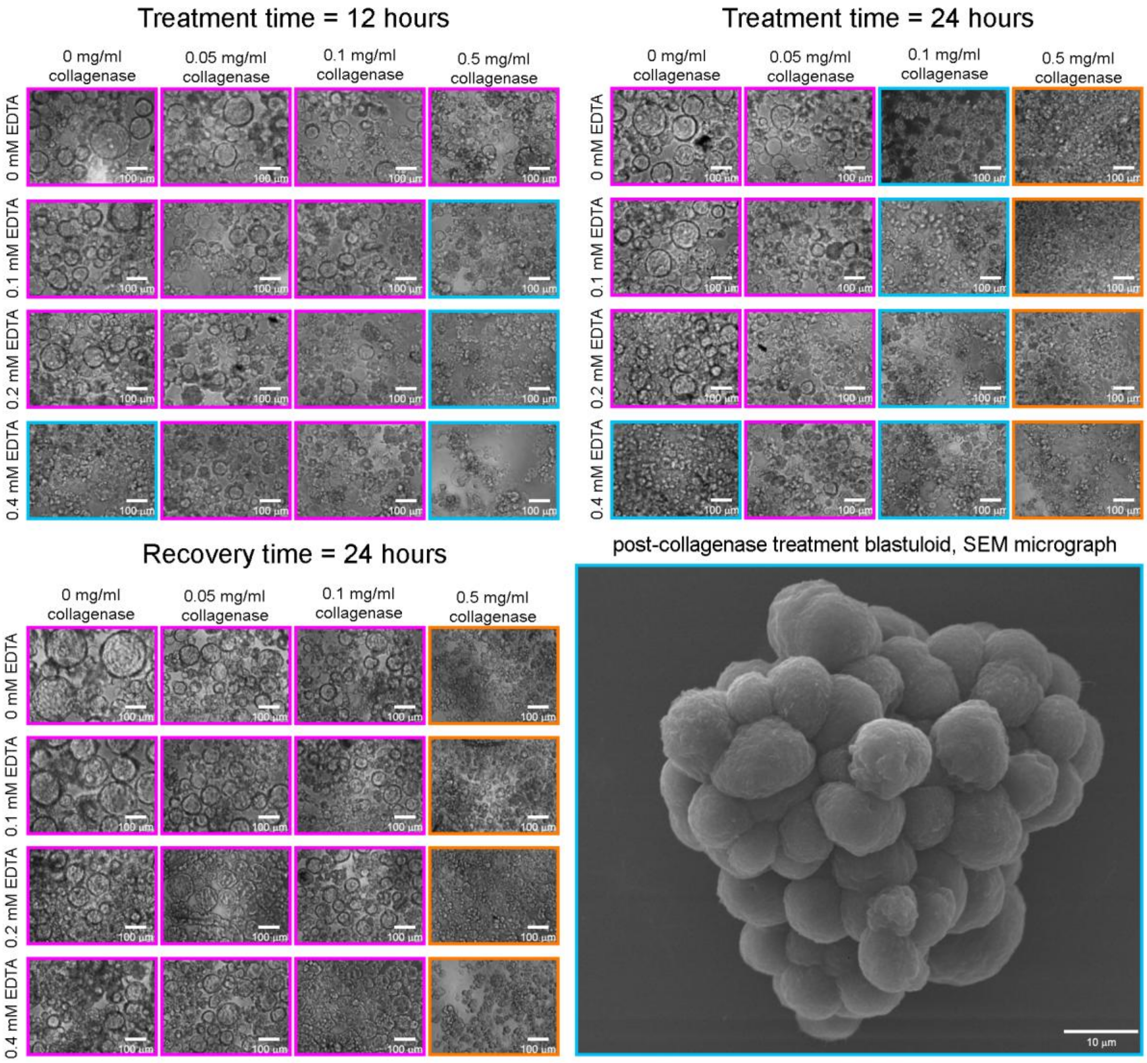

SI Fig. 4. Collagenase treatments and recovery (independent as well as in combination with EDTA treatments) over different concentrations and varying treatment times, suggesting that perturbing the cell-matrix interactions, in addition to disrupting cell-cell junctions, exerts an additive effect on transitions between different conformational states. Color scale: orange indicates single cells, cyan indicates moruloids, and magenta indicates blastuloids.

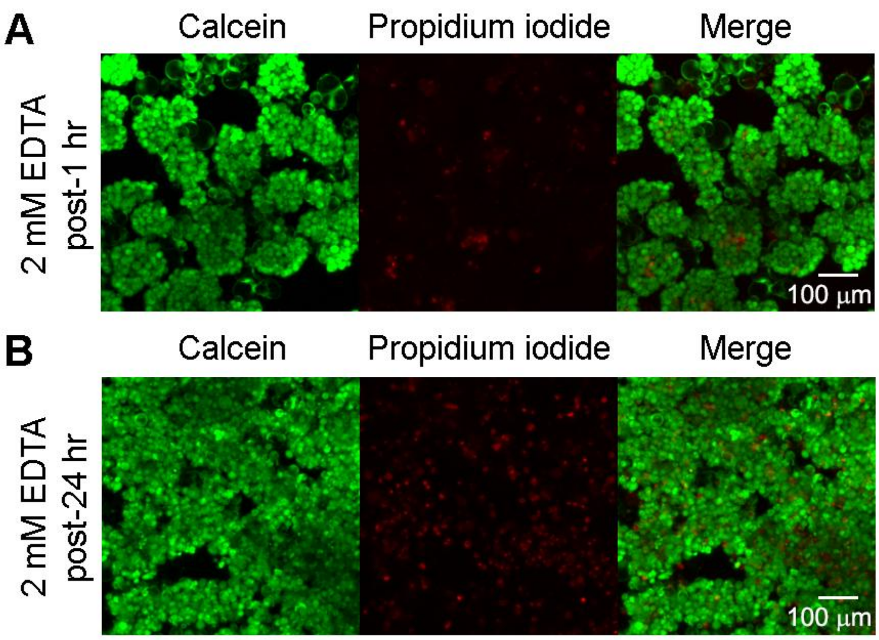

SI Fig. 5. Cell viability under (**A**) 1 hour-long and (**B**) 24 hours-long EDTA treatments, showing that the disaggregation is not due to cell death.

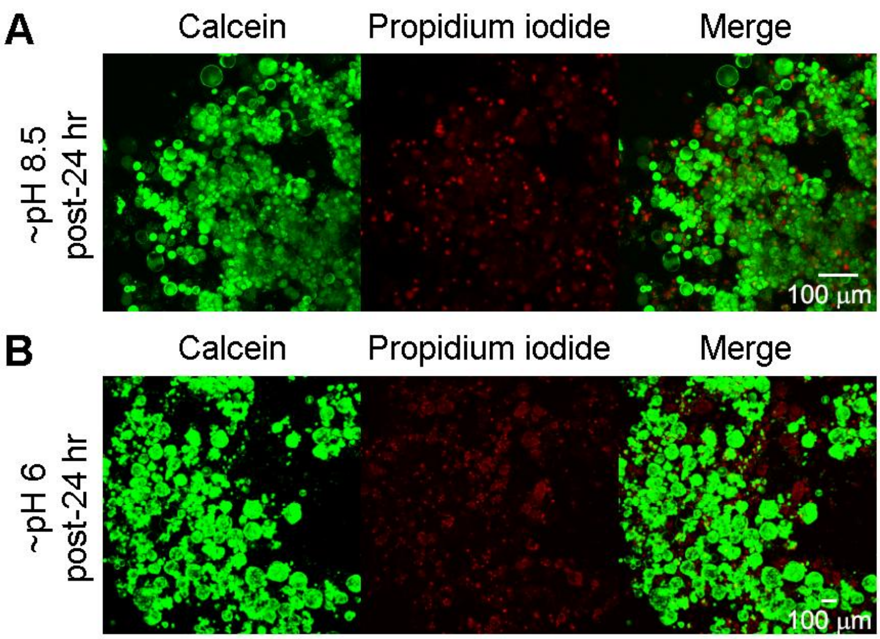

SI Fig. 6. Cell viability under (**A**) basic pH and (**B**) acidic pH treatments, showing that conformational transitions are not due to loss of cell viability.

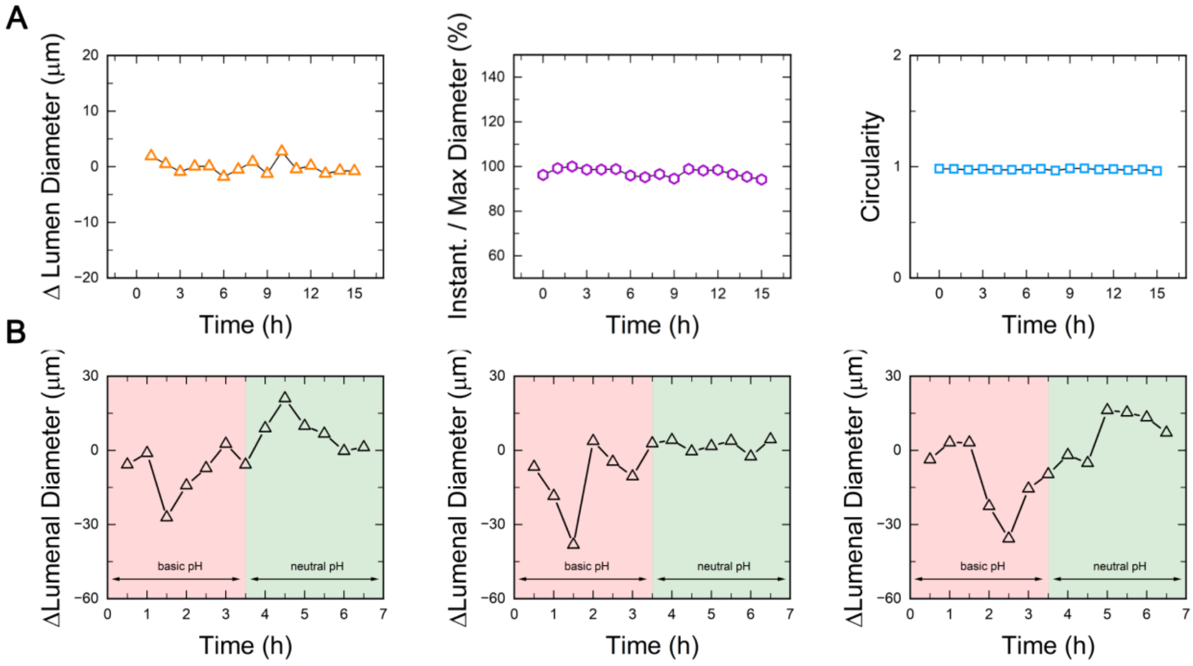

SI Fig. 7. Time trace analysis of the lumenal dimensions for (A) acidic pH-treated blastuloid and (B) blastuloids undergoing a basic pH-induced collapse, followed by recovery at pH ~7.4.

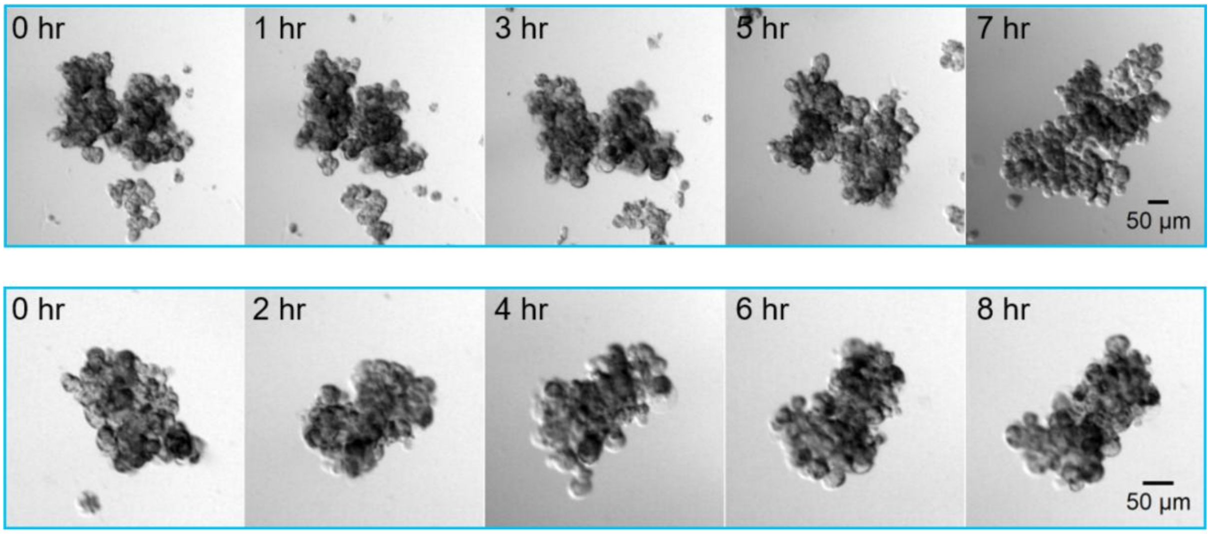

SI Fig. 8. Time-lapse imaging of two OVCAR4 spheroids in suspension culture. Color scale: cyan indicates non-lumenized aggregates.

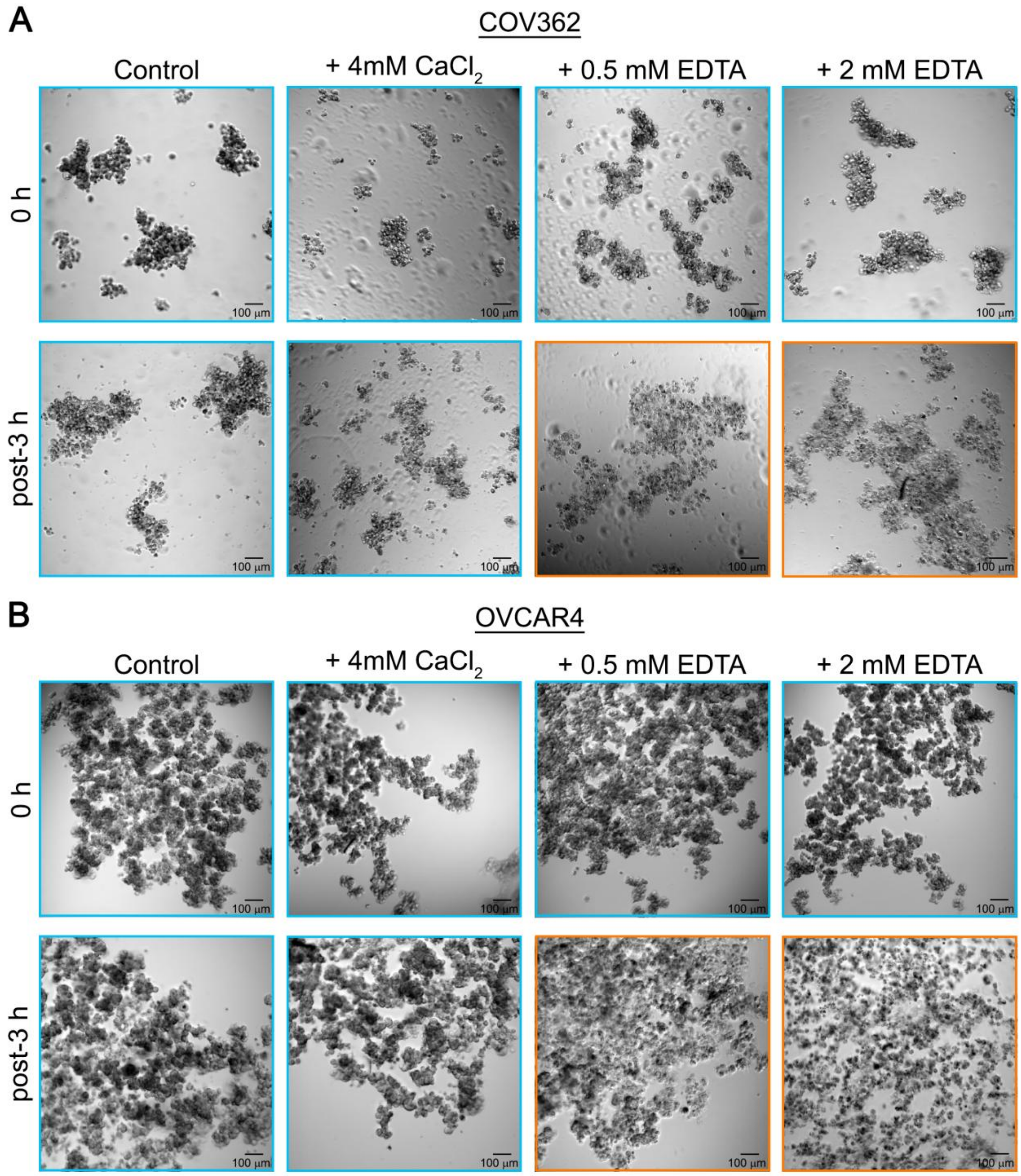

SI Fig. 9. Response of cellular collectives formed by (A) COV362 and (B) OVCAR4 cells to calcium supplementation ($CaCl_2$ treatment) and chelation (EDTA treatment), which phenocopy the responses exhibited by OVCAR3. Color scale: orange indicates single cells and cyan indicates non-lumenized aggregates.

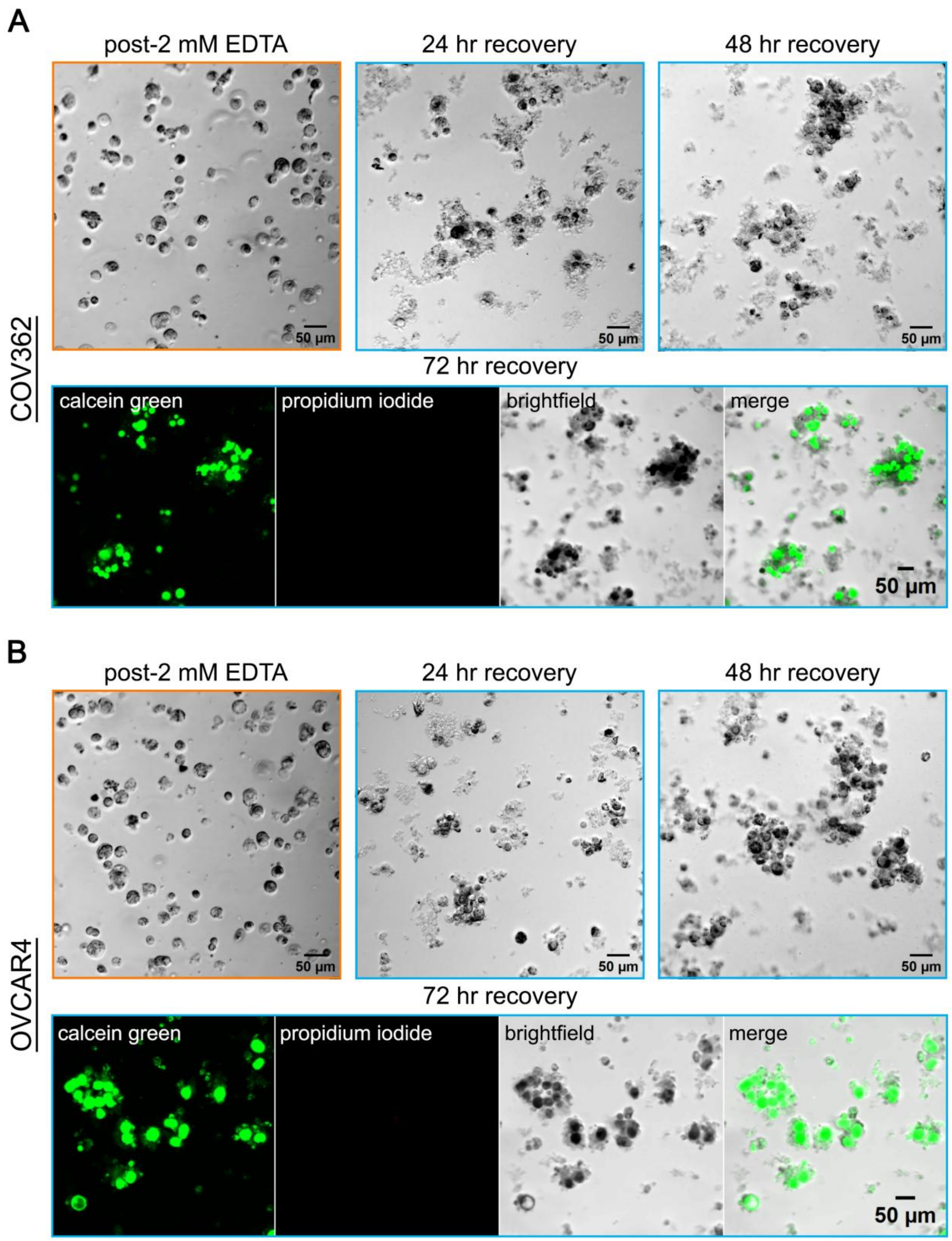

SI Fig. 10. Recovery and viability of aggregate ensembles generated by self-assembly of dispersed single cell suspensions which are formed after 2 mM EDTA treatment-induced collapse of (A) COV362 and (B) OVCAR4 collectives. Color scale: orange indicates single cells and cyan indicates non-lumenized aggregates.

Supplementary videos captions

Supplementary video 1: Timelapse imaging of a blastuloid composed of constitutively GFP-expressing cells exhibiting the periodic conformational transitions.

Supplementary video 2: Timelapse imaging of blastuloids composed of OVCAR-3 cells carrying a constitutively expressing construct of E-Cadherin fused to GFP, which localizes to the cell-cell junctions. This allows real-time visualization of both cell morphologies and cell junctional rearrangements during the conformational transitions.

Supplementary video 3: Timelapse imaging of a blastuloid undergoing complete collapse due to loss of cell-cell junctional integrity under 2 mM EDTA treatment.

Supplementary video 4: Timelapse imaging of a blastuloid comprising cells expressing the GFP-E-cad fusion construct, maintained under pH ~ 6, wherein the acidic environment abrogates periodic conformational transitions and instead, arrests the blastuloid in the maximally lumenized state.

Supplementary video 5: Comparison of (*left*) blastuloids undergoing basic pH-induced collapse of lumens (without CO_2 supply) against (*right*) the same blastuloids recovering their lumenized forms under conditions of environmental pH ~ 7.4 (restored CO_2 supply).

Supplementary video 6: Timelapse imaging of a blastuloid composed of constitutively GFP-expressing cells, embedded within a 3D agarose microparticles-based jammed microgel system. Here, the blastuloid continues to exhibit its characteristic periodic conformational transitions, albeit now within a deformable yet self-healing soft viscoelastic 3D matrix.

Supplementary video 7: Timelapse imaging of an OVCAR4 aggregate in liquid suspension, showing the dynamic nature of cells comprising the ensemble structure.
